# Molecular Mechanisms for the Carnosine-Induced Activation of Muscle–Brain Interaction

**DOI:** 10.3390/nu15061479

**Published:** 2023-03-19

**Authors:** Asuka Ishibashi, Miyako Udono, Mikako Sato, Yoshinori Katakura

**Affiliations:** 1 Graduate School of Bioresources and Bioenvironmental Sciences, Kyushu University, Fukuoka 819-0395, Japan; 2 Faculty of Agriculture, Kyushu University, Fukuoka 819-0395, Japan; mudono@grt.kyushu-u.ac.jp; 3 R&D Center, NH Foods, Ltd., Tsukuba 300-2626, Japan; m.satou@nipponham.co.jp

**Keywords:** carnosine, muscle–brain interaction, gut–brain interaction, neuronal cells, miRNAs, exosomes

## Abstract

Carnosine is known to improve brain function. The molecular basis for the carnosine-mediated interaction between intestinal cells and neuronal cells is that carnosine acts on intestinal cells and stimulates exosome secretion, which can induce neurite outgrowth in neuronal cells. This study aimed to infer the carnosine-mediated interaction between muscle cells and neuronal cells. The results revealed that carnosine induces muscle cell differentiation, as well as the secretion of exosomes and myokines that can act on neuronal cells. Carnosine acts not only on intestinal cells but also on muscle cells, stimulating the secretion of secretory factors including exosomes that induce neurite outgrowth in neuronal cells, as well as myokines known to be involved in neuronal cell activation. As the miRNAs in exosomes secreted from intestinal cells and muscle cells upon carnosine treatment are different, it could be assumed that carnosine acts on each cell to interact with neuronal cell through separate factors and mechanisms.

## 1. Introduction

Carnosine (*β*-alanyl-l-histidine) is an imidazole dipeptide (IDPs) that exerts various physiological functions in humans, including pH-buffering action and antioxidant activity [1]. Meats are known to contain more IDPs than other foods, and among these meats, chicken breast has the highest IDP content. Among the various functions of carnosine, many studies have recently been reported, especially on the regulation of brain functions. Studies using Alzheimer’s model mice and double-blind randomized controlled trials have shown that carnosine can improve memory function [2], that is, the ability to activate the gut–brain interaction. The mechanisms underlying carnosine-mediated activation of the gut–brain interaction have been shown to include increased cerebral blood flow and decreased levels of inflammatory cytokines in the blood [3,4]. However, carnosine is degraded in the blood soon after ingestion, and the mediators that mediate carnosine functionality have not been identified. Therefore, by employing an in vitro cell culture system, we showed the possibility that carnosine acts on intestinal cells (Caco-2 cells), which in turn secrete exosomes that can promote neurite outgrowth in neuronal cells (SH-SY5Y cells) and reported this as a new molecular mechanism for the carnosine-induced interaction between intestinal cells and neuronal cells [5]. Through integrated analysis of miRNAs in exosomes derived from carnosine-treated Caco-2 cells and mRNAs in SH-SY5Y cells treated with exosome derived from carnosine-treated Caco-2 cells, miRNAs and their target genes mediating carnosine function were previously identified. Exosomes have been pointed out as a potential marker for various diseases, including cancer, and a cancer diagnostic system based on blood exosomes is being developed, but this is one of the few examples that show the potential of exosomes to mediate food functionality and even organ–organ interactions with food.

Previous reports have shown that carnosine is present in particularly high concentrations and at millimolar levels in skeletal muscle and olfactory bulb [1]. In recent years, muscle has received increasing attention as an endocrine tissue. Recent reports suggest that myokines, such as brain-derived growth factor (BDNF) and IGF-I secreted by muscle cells, are involved in the interaction with neuronal cells, and may mediate the beneficial effects of physical exercise [6]. In other words, muscle cells have been suggested to crosstalk with various cells via myokine secretion. More recently, it has also become clear that not only myokine but also exosomes are involved in interactions between various cells. In this study, we tried to examine the possibility that carnosine induces muscle cells to secrete exosomes that interact with neuronal cells.

## 2. Materials and Methods

### 2.1. Cell Culture and Reagents

Murine skeletal myoblasts C2C12 (Riken Bioresource Center, Tsukuba, Japan) and human neuronal cells SH-SY5Y (ATCC, Manassas, VA, USA) were cultured in DMEM (Nissui, Tokyo, Japan) containing 10% heat-inactivated fetal bovine serum (FBS, Capricorn Scientific GmbH, Ebsdorfergrund, Germany) at 37 °C in 5% CO_2_. Carnosine, AICAR and retinoic acid (RA) were purchased from FUJIFILM Wako Pure Chemical Co. (Osaka, Japan).

### 2.2. Differentiation of C2C12

C2C12 cells (2.0 × 10^5^ cells/mL at 6-well dish) were cultured for 48 h, the medium was replaced with DMEM containing 2% horse serum (HS) (Thermo Fisher Scientific, Waltham, MA, USA) to induce differentiation, and carnosine (1 to 50 mM) was added where indicated. After another 24 h, the medium was replaced with DMEM containing 2% HS and carnosine was added. After that, the medium was changed, carnosine was added every 2 days, and cells were used for the experiment on the 10th day after seeding.

### 2.3. Reverse Transcriptase-Quantitative Polymerase Chain Reaction (RT-qPCR)

Total RNA was prepared using the High Pure RNA Isolation Kit (Roche Diagnostics Gmbh, Mannheim, Germany). RT-qPCR was performed using GoTaq 1-Step RT-qPCR System (Promega, Madison, WI, USA) on Thermal Cycler Dice Real Time System TP-800 (Takara Shiga, Japan) as described previously [5]. The PCR primer sequences are listed in Table S1. For normalization against reference genes, Ct values of target genes were normalized by the CT values of *β*-actin and analyzed using the ΔΔCT method [7]. All reactions were performed in triplicate. Melting point analysis was performed to confirm the presence of a single PCR product. The specificity of the PCR reaction was confirmed by amplification plots and dissociation curve data for each gene (Figure S1). The efficiency of the reaction was confirmed based on Ct values of 25–35 for the amplification reaction. The variability of the results was statistically evaluated based on the variability of the results of experiments in triplicate.

### 2.4. Fluorescent Immunocytochemistry

After the cells were fixed with a 4% paraformaldehyde for 15 min at 25 °C, they were treated with blocking buffer (PBS containing 5% normal goat serum and 0.3% Triton X-100) for 1 h at 25 °C. After washing the cells, the cells were treated with primary antibodies (rabbit anti-fast myosin skeletal heavy chain ab91056, Abcam, Cambridge; mouse anti-slow skeletal myosin heavy chain, ab11083, Abcam) at 4 °C overnight under shielded light. After washing the cells, the cells were treated with secondary antibodies (goat anti-rabbit Alexa Fluor 647, ab150079, Abcam; Goat anti-mouse Alexa Fluor 488, 115-547-003, Jackson ImmunoResearch, West Grove, PA, USA) for 1 h under shielded light. After washing the cells, the cells were treated with Hoechst 33342 solution (Dojindo, Kumamoto, Japan) for 20 min, and the stained cells were analyzed using an IN Cell Analyzer 2200 (Cytiva, Tokyo, Japan).

After the differentiated cells were fixed and blocked in the same manner, they were reacted with mouse anti-slow skeletal myosin heavy chain antibody and mitochondria-specific rabbit anti-TOMM20 antibody (ab186735, Abcam), and then stained with anti-mouse Alexa Fluor 488 and anti-rabbit Alexa Fluor 647, respectively. As described above, slow muscle and mitochondria were stained with anti-slow skeletal myosin heavy chain antibody and anti-TOMM20 antibody, respectively. The total mitochondrial area in slow muscle was measured using IN Cell Investigator High-content image analysis software (Cytiva). The analysis showed 200–500 cells per sample and 10–30 mitochondria per cell.

### 2.5. Quantitative Evaluation of Neurite Length

SH-SY5Y cells were fixed with 4% paraformaldehyde for 15 min. After washing the cells, the cells were treated with a blocking buffer (1 × PBS, 5% goat serum, and 0.3% Triton X-100) for 1 h, and subsequently with Milli-Mark Pan Neuronal Marker (Merck Millipore, Billerica, MA, USA) at 25 °C overnight. After washing the cells, the cells were stained with Alexa Fluor 555 goat anti-rabbit IgG antibody (Thermo Fisher Scientific, Inc., Tokyo, Japan) and Hoechst 33342, and neurite outgrowth was quantitatively determined using the IN Cell Analyzer 2200 (Cytiva, Tokyo, Japan), as previously described [8].

### 2.6. Exosome Isolation and Treatment

First, C2C12 cells (2.0 × 10^5^ cells/mL) were cultured in DMEM containing 2% Exosome-depleted FBS (System Bioscience, Mountain View, CA, USA) and carnosine (10 to 50 mM) for 10 days while changing the medium every 2 days. MagCapture Exosome Isolation PS Kit Ver. 2 (FUJIFILM Wako Pure Chemical Corp.) was used to isolate exosomes from the media of C2C12 cells as described previously [8]. The amount of exosome used for each experiment was prepared as protein equivalent as measured by MicroBCA Protein Assay Kit (Thermo Fisher Scientific Inc.). SH-SY5Y cells (2.0 × 10^5^ cells/mL) were treated with exosomes (equivalent to 90 ng/well) for 24 h [5,9].

### 2.7. miRNA Microarray Assay

The expression profiles of miRNAs in exosomes were evaluated using microarray analysis with a 3D-Gene Human miRNA Oligo chip (Toray, Kanagawa, Japan). miRNA preparation and subsequent operations were outsourced to Kamakura Techno-Sciences, Inc. (Kanagawa, Japan). After global normalization of miRNA expression levels, we calculated the ratios of each gene for comparison between control and experimental samples. We then established criteria for regulated genes: (upregulated genes) ratio ≥ 2.0-fold [10]. The miRNA target genes were predicted using TargetScan (https://www.targetscan.org/vert_80/, accessed on 1 February 2022). We then used tools and data provided by the Database for Annotation, Visualization, and Integrated Discovery (DAVID, http://david.abcc.ncifcrf.gov, accessed on 20 February 2022) to determine significantly enriched pathways [8,11,12].

### 2.8. Statistical Analysis

Experiments were repeated at least three times, and the representative data are shown. The results are shown as the mean ± standard error. Multiple comparisons between groups were performed using one-way ANOVA with Tukey’s post-hoc test. Statistical significance was defined as *p* < 0.05 when compared to control (* *p* < 0.05; ** *p* < 0.01; *** *p* < 0.001).

## 3. Results

### 3.1. Effects of Carnosine on Muscle Cell Differentiation

**Figure 1:**
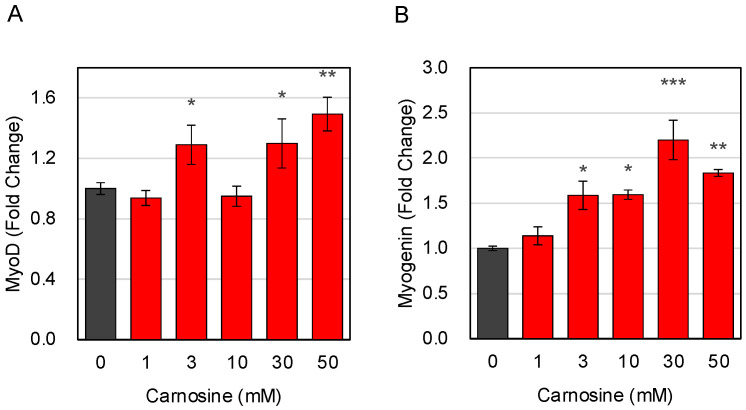
Expression of differentiation marker gene. C2C12 cells were differentiated in the presence of indicated amount of carnosine. Expression of muscle differentiation marker genes of MyoD (**A**) and Myogenin (**B**) was quantitatively determined by RT-qPCR. Multiple comparisons between groups were performed using one-way ANOVA with Tukey’s post-hoc test. Statistical significance was defined as *p* < 0.05 when compared to control (* *p* < 0.05; ** *p* < 0.01; *** *p* < 0.001).

The effect of carnosine on the induction of differentiation was evaluated in differentiating C2C12 cells. From 1 to 50 mM carnosine was added to C2C12 cells and cultured, but no particular difference in cell viability was observed. No significant toxicity was observed after incubation with high concentrations of carnosine (Figure S2). The mRNA expression levels of MyoD and Myogenin, which are genes related to muscle cell differentiation, were evaluated by RT-qPCR. MyoD is expressed from the proliferative phase of myoblasts to the early maturation phase of myotubular cells, whereas Myogenin is expressed from myocytes to the late maturation phase of myotubular cells. Both MyoD and Myogenin expressions were significantly increased in C2C12 cells differentiated in the presence of carnosine (Figure 1A,B), suggesting that carnosine can induce C2C12 cell differentiation.

### 3.2. Effects of Carnosine on Muscle Fiber Type Change

**Figure 2:**
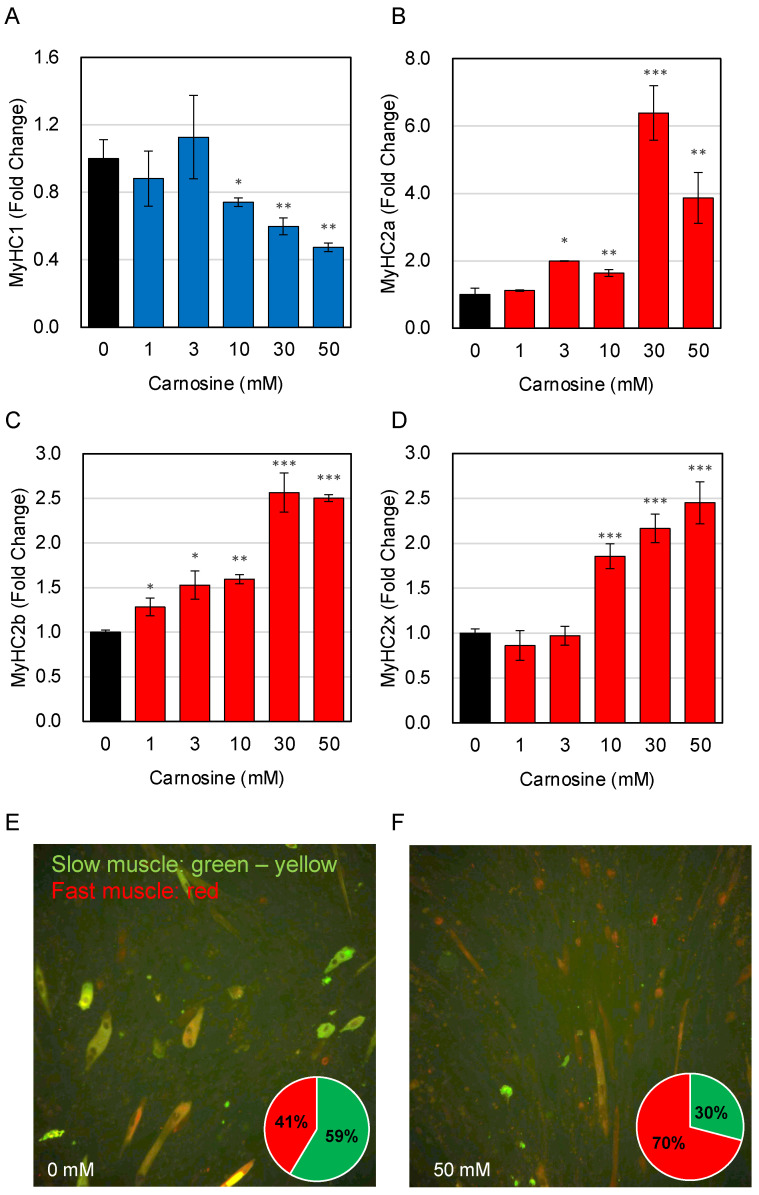
Effects of carnosine on muscle fiber type change. C2C12 cells were differentiated in the presence of indicated amount of carnosine. Expression of slow and fast muscle marker genes of MyHC1 (**A**), MyHC2a (**B**), MyHC2b (**C**), and MyHC2x (**D**) was quantitatively determined by RT-qPCR. Effects of carnosine on muscle fiber type was evaluated by immunocytochemitory using antibodies (rabbit anti-fast myosin skeletal heavy chain (red) and mouse anti-slow skeletal myosin heavy chain (green to yellow)). Relative abundance of fast and slow muscle was determined by using IN Cell Analyzer 2200 ((**E**), non-treat; (**F**), 50 mM carnosine treatment). Multiple comparisons between groups were performed using one-way ANOVA with Tukey’s post-hoc test. Statistical significance was defined as *p* < 0.05 when compared to control (* *p* < 0.05; ** *p* < 0.01; *** *p* < 0.001).

We examined the effects of carnosine on changes in muscle fiber type. A slow muscle marker (MyHC1) and fast muscle markers (MyHC2a, MyHC2x, and MyHC2b) were used to verify mRNA expression levels in C2C12 cells after differentiation in the presence of carnosine using RT-qPCR. The results revealed that MyHC1 levels was decreased and MyHC2a, MyHC2x, and MyHC2b levels were increased in C2C12 cells differentiated in the presence of carnosine (Figure 2A–D). The changes in MyHC expression indicated that carnosine induces fast-twitch synthesis in C2C12 cells.

Next, fluorescent immunocytochemistry using anti-fast myosin skeletal heavy chain antibody and anti-slow skeletal myosin heavy chain antibody were used to analyze carnosine-induced changes in the muscle fiber type (Figure 2E,F). Fast-twitch muscles appear to be stained red, while slow-twitch muscles appear to be stained green to yellow. These results also showed that carnosine treatment increased the percentage of fast-twitch myotubes.

### 3.3. Effects of Carnosine on the Expression of Factors Secreted from Myotube Cells

**Figure 3:**
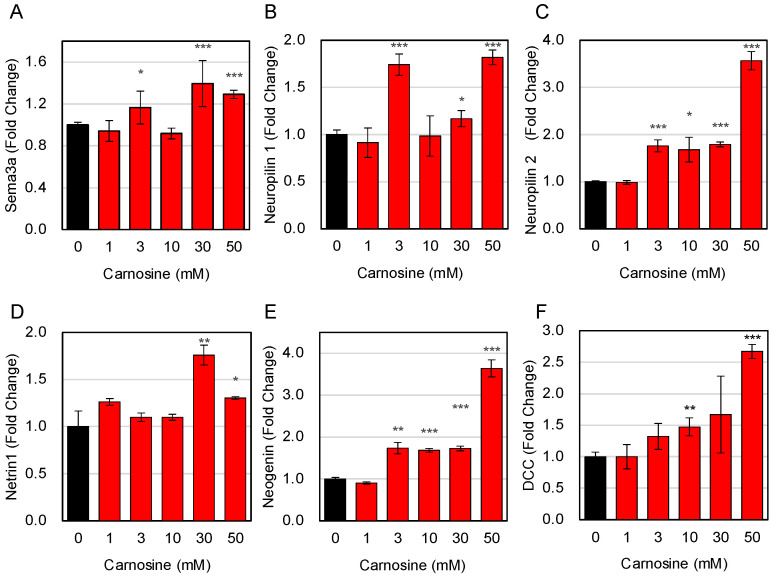
Effects of carnosine on the expression of slow and fast-related factors in myotube cells. C2C12 cells were differentiated in the presence of indicated amount of carnosine. Expression of slow and fast muscle-related factor genes of Sema3a (**A**), Neuropilin 1 (**B**), Neuropilin 2 (**C**), Netrin 1 (**D**), Neogenin (**E**), and DCC (**F**) was quantitatively determined by RT-qPCR. Multiple comparisons between groups were performed using one-way ANOVA with Tukey’s post-hoc test. Statistical significance was defined as *p* < 0.05 when compared to control (* *p* < 0.05; ** *p* < 0.01; *** *p* < 0.001).

Here, we focused on Sema3A, which contributes to slow muscle differentiation [13], and Netrin-1, which contributes to fast muscle differentiation [14] and examined their expression changes upon carnosine treatment using RT-qPCR. The results showed that carnosine treatment increased the expression of both Sema3A and Netrin-1 (Figure 3A,D). In addition, the expression levels of neuropilin 1 and neuropilin 2, receptors for Sema3A, and Neogenin and deleted in colorectal cancer (DCC), receptors for Netrin-1, were evaluated by RT-qPCR. The results showed that carnosine treatment increased the expression of all receptors (Figure 3B,C,E,F). Unlike MyHC expression, as for myotube-derived secreted factors and their receptors, the expression of both slow and fast muscle-related genes was enhanced.

### 3.4. Effects of Carnosine on Mitochondria in Myotube Cells

**Figure 4:**
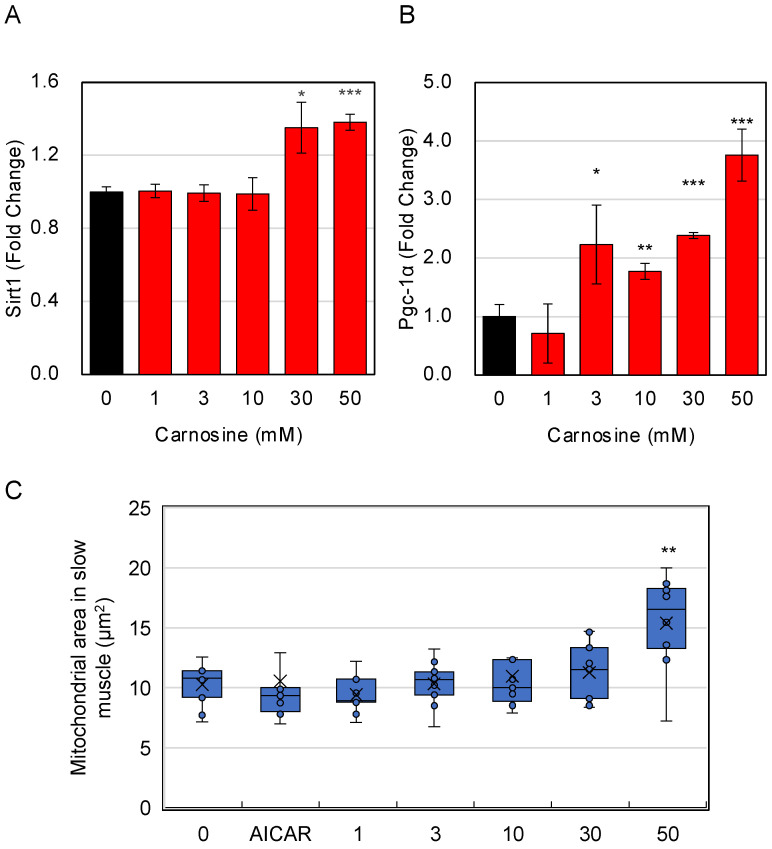
Effects of carnosine on mitochondria in myotube cells. Effects of carnosine on the expression of mitochondria-related genes in myotube cells. C2C12 cells were differentiated in the presence of the indicated amount of carnosine. Expression of mitochondria-related genes of Sirt1 (**A**), and Pgc-1α (**B**) was quantitatively determined by RT-qPCR. (**C**) The size of mitochondria in myotubes was determined by using IN Cell Analyzer 2200. As a control experiment, C2C12 cells were treated with 250 µM AICAR, the AMPK activator for 3 days. Multiple comparisons between groups were performed using one-way ANOVA with Tukey’s post-hoc test. Statistical significance was defined as *p* < 0.05 when compared to control (* *p* < 0.05; ** *p* < 0.01; *** *p* < 0.001).

Since carnosine was suggested to have an effect not only on fast-twitch muscles but also on slow-twitch muscles, we tested its effect on mitochondrial activity, a characteristic of slow-twitch muscles. Therefore, we focused on Pgc-1α, the master gene for mitochondrial biogenesis, and its activator, the longevity gene Sirt1 [15], and examined the effect of carnosine on its expression. The results showed that carnosine enhanced the expression of Sirt1 and Pgc-1α (Figure 4A,B), suggesting that carnosine can induce the biogenesis of mitochondria in myotubes. Therefore, we used the IN Cell Analyzer 2200 to examine whether carnosine treatment changed the size of mitochondria in the cell. Although the effect of AICAR, an AMPK activator, on increasing the size of mitochondrial cannot be observed, the results showed that the size of the mitochondria in myotubes increased significantly with carnosine treatment (Figure 4C). Optimization of AICAR treatment is considered to be necessary in the future study.

### 3.5. Effects of Carnosine on the Interaction between Muscle Cells and Neuronal Cells

**Figure 5:**
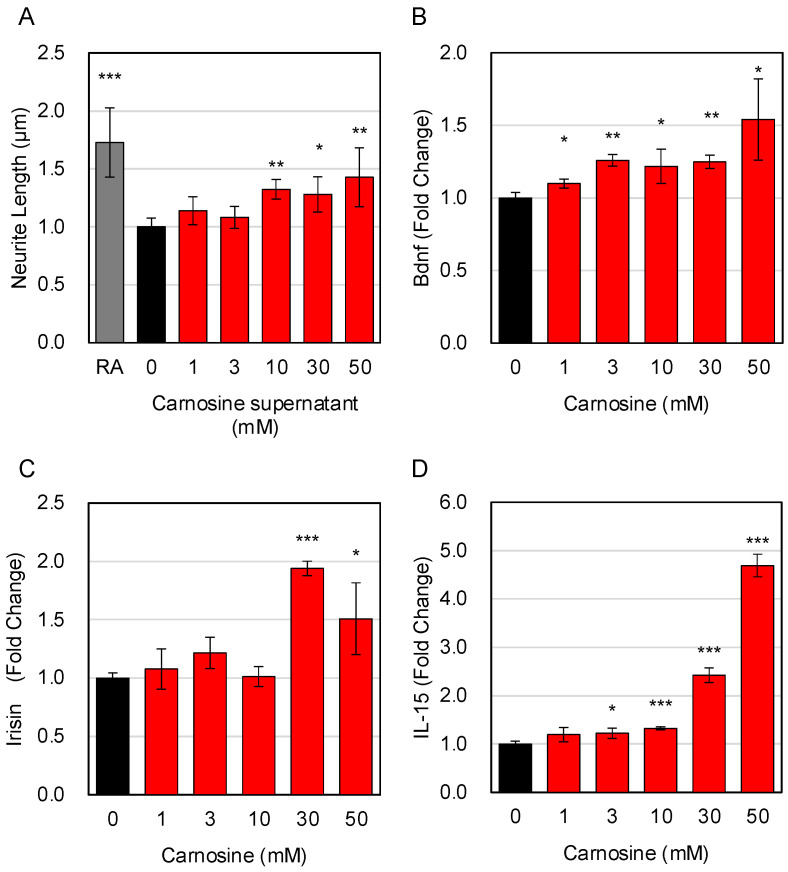
Effects of carnosine on the interaction between muscle cells and neuronal cells. C2C12 cells were differentiated in the presence of the indicated amount of carnosine. Culture supernatants of carnosine-treated C2C12 cells were added to SH-SY5Y cells, and neurite outgrowth in SH-SY5Y cells was evaluated using IN Cell Analyzer 2200. As a positive control, SH-SY5Y cells were cultured with 10 μM of retinoic acid (RA) for 1 day (**A**). The effect of carnosine on the expression of myokine (Bdnf (**B**); Irisin (**C**); IL-15 (**D**)) was quantitatively determined by RT-qPCR. Multiple comparisons between groups were performed using one-way ANOVA with Tukey’s post-hoc test. Statistical significance was defined as *p* < 0.05 when compared to control (* *p* < 0.05; ** *p* < 0.01; *** *p* < 0.001).

Next, we examined the possibility that carnosine may induce the interaction between muscle cells and neuronal cells. Therefore, we first prepared culture supernatants of carnosine-treated C2C12 cells and evaluated whether the addition of the supernatants to SH-SY5Y, a neuronal cell, induced neurite outgrowth in neuronal cells. Since retinoic acid (RA) is known to induce neurite outgrowth in SH-SY5Y cells by direct addition, it was used as a positive control. The results showed that culture supernatant of carnosine-treated C2C12 cells could induce neurite outgrowth in SH-SH5Y (Figure 5A). Next, we evaluated the effect of carnosine on the expression of myokine, a muscle-derived factor, in C2C12 cells, which seems to be involved in the interaction between muscle cells and neuronal cells. The results showed that the expression of Bdnf, Irisin, and IL-15, known as myokines, in C2C12 cells was significantly enhanced upon carnosine treatment (Figure 5B–D). The enhanced expression of these myokines in muscle cells following carnosine treatment may form part of the molecular basis for the carnosine-induced interaction between muscle cells and neuronal cells.

**Figure 6:**
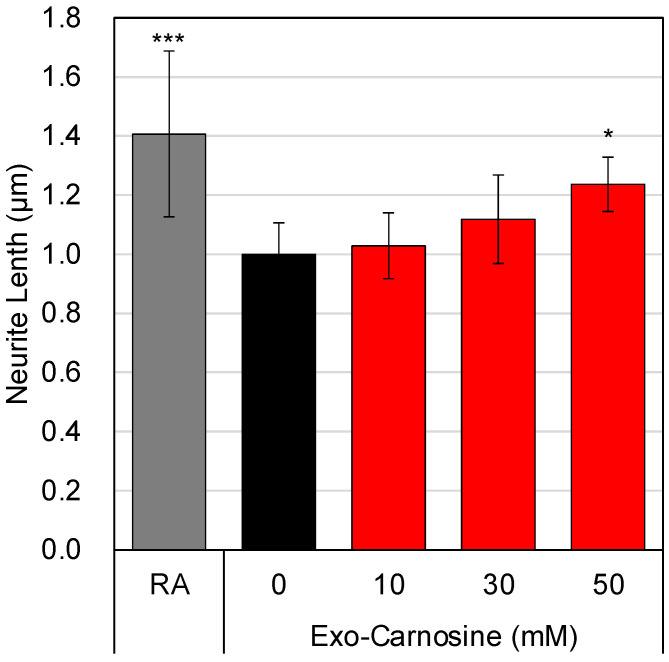
Exosome derived from carnosine-treated C2C12 cells activated neurite outgrowth in SH-SY5Y cells. Exosomes were prepared from carnosine-treated C2C12 cells by MagCapture Exosome Isolation kit and added to SH-SY5Y cells. As a positive control, SH-SY5Y cells were cultured with 10 μM of RA for 1 day. Neurite outgrowth in SH-SY5Y cells was evaluated by using IN Cell Analyzer 2200. Multiple comparisons between groups were performed using one-way ANOVA with Tukey’s post-hoc test. Statistical significance was defined as *p* < 0.05 when compared to control (* *p* < 0.05; *** *p* < 0.001).

We further focused on muscle cell-derived exosomes as another molecular basis for the carnosine-induced interaction between muscle cells and neuronal cells. Therefore, we added exosomes prepared from the supernatant of carnosine-treated C2C12 cells, using the MagCapture Exosome Isolation kit, to SH-SY5Y cells and evaluated the neurite outgrowth induced by exosomes. The results showed that carnosine-treated C2C12 cell-derived exosomes significantly elongated the neurites in SH-SY5Y cells (Figure 6). The exosomes secreted from muscle cells in response to carnosine are also thought to form part of the molecular basis for the carnosine-induced interaction between muscle cells and neuronal cells.

### 3.6. Molecular Basis for the Carnosine-Induced Interaction between Muscle Cells and Neuronal Cells

Next, we focused on miRNAs in muscle cell-derived exosomes as the molecular basis for the carnosine-induced interaction between muscle cells and neuronal cells. Therefore, we focused on miRNAs in C2C12 cell-derived exosomes and identified seven miRNAs whose expression was significantly changed upon carnosine treatment (Table 1). After estimating the target genes of these miRNAs, functional annotation and KEGG pathway analyses were performed using DAVID (Table 1 and Table 2). First, functional annotation analysis revealed that many genes with functions related to brain development, nervous system development, dendrites, and synapses were significantly affected (Table 1). Furthermore, the results of the KEGG pathway analysis indicated that pathways related to long-term potentiation, axon-guidance, and cAMP signaling pathway were significantly affected (Table 2). These results showed that carnosine-treated muscle cell-derived exosomes can interact with neuronal cells and suggested that these exosomes may regulate the activity and/or function of neuronal cells.

## 4. Discussion

Skeletal muscle is the most abundant tissue in mammals and is characterized by contraction and extension. Muscle fibers are classified into four types: MyHC1 (Type I), MyHC2a (Type IIA), MyHC2x (Type IIX) and MyHC2b (Type IIB) [16]. Type I and Type IIA are known to be oxidative fibers containing mitochondria and myoglobin. Type I fiber is also known as a slow fiber. On the other hand, Type IIB is known to be glycolytic fibers. Oxidative fiber is known to contribute to weight loss and health promotion, and increasing oxidative muscle has become a goal of functional food development. Recently, types of food, such as various natural plant extracts including catechins and polyphenols, have been shown to regulate muscle fiber types and have beneficial effects on health [17]. In this study, the effects of carnosine, another food ingredient derived from meat, on muscle fiber type change and muscle cell functions were examined using C2C12 cells. The results revealed that carnosine induces C2C12 differentiation, fast muscle at the level of MyHC expression, slow and fast muscle at the level of secreted myokines, and slow muscle at the level of mitochondrial synthesis-promoting levels, indicating that carnosine acts on C2C12 in various ways depending on myofiber markers. Carnosine appears to induce both slow and fast-twitch fibers, depending on the environment in which C2C12 is located and how it is phenotypically observed. The detailed mechanisms and phenotypes of carnosine-induced slow and fast-twitch fiber induction need to be clarified in future studies. Since there have been few functional analyses of carnosine using C2C12 cells [18,19], we believe that novel functionalities of carnosine through muscle cells can be found.

In our previous studies, we have shown by double-blind randomized controlled trials that carnosine improves memory function [2,20], and we have also hypothesized that part of the molecular basis for this improvement is that intestinal cells encountering carnosine secrete exosomes that induce neurite outgrowth in neuronal cells [5]. As exercise is effective in improving cognitive function [21] and enhances carnosine synthesis in skeletal muscles [22], the purpose of this study was to clarify the carnosine-induced interaction between muscle cells and neuronal cells. Previous studies have found that carnosine acts on intestinal cells and stimulates the secretion of exosomes that induce the neurite outgrowth in neuronal cells [4]. This study also examined the possibility of carnosine-induced interaction between muscle cells and neuronal cells via the resulting secreted factor. Various analyses have revealed that carnosine promotes and induces muscle cell differentiation and the secretion of exosomes and cytokines, which are the mediators of interaction between muscle cells and neuronal cells. With regard to exosomes in particular, it was possible to consider that carnosine induced secretion of exosomes that induce the neurite outgrowth in neuronal cells rather than enhanced exosome secretion from muscle cells, i.e., carnosine induced a qualitative rather than quantitative change in exosomes. The fact that the miRNAs in the exosomes that carnosine induces secretion of from intestinal cells and the exosomes that it induces secretion of from muscle cells are different indicates that carnosine acts on the intestinal cells and muscle cells separately and induces the secretion of different exosomes to interact with neuronal cells.

In this study, we demonstrated that muscle cell-derived exosomes induced the neurite outgrowth in neuronal cells. Although there have been studies on the effects of adipocyte-derived exosomes on skeletal muscle [23] and the involvement of exosomes in muscle–kidney interactions [24], there are few reports on the functionality of exosomes produced by muscle cells [25]. In particular, results so far suggest that carnosine may induce the interaction between intestinal cells, muscle cells and neuronal cells via exosomes. This study may represent an important result regarding the elucidation of the functionality of exosomes.

Although this study focused on in vitro studies and showed some of the molecular basis of carnosine-induced interactions between muscle cells and neuronal cells, there are still limitations, and future experiments should be conducted to clarify the activity and functionality of carnosine for muscle–brain interactions.

## Figures and Tables

**Table 1:** Functional annotation of genes regulated by miRNAs.

miRNA	Ratio	Functional Annotation	Enrichment Score
miR-6240	4.00	brain development	9.305
		nervous system development	7.446
		central nervous system neuron differentiation	6.410
		dendrite	4.859
		synapse	4.590
		central nervous system neuron development	3.683
		neuron spine	3.537
		synaptic signaling	3.423
miR-1983	2.57	central nervous system development	11.885
		nervous system development	10.593
		dendrite	9.141
		synaptic signaling	8.154
		synapse	5.675
		axon part	5.199
		learning or memory	4.093
miR-3963	2.37	nervous system development	6.118
		synapse	4.202
		synapse part	3.550
		synaptic signaling	3.193
miR-3968	2.25	nervous system development	10.328
		synapse	7.524
		brain development	6.161
		dendrite	5.653
		axon	3.739
		axon extension	3.459
miR-125a-3p	2.23	synapse	16.259
		nervous system development	11.463
		axon	8.528
		synaptic signaling	8.131
		brain development	5.358
		neuron spine	5.216
		learning or memory	3.978
		dendrite development	3.651
miR-6366	2.10	nervous system development	10.013
		synapse	8.803
		synaptic signaling	8.568
		brain development	6.534
		axon	6.077
		dendrite	5.448
		synapse part	3.737
		central nervous system neuron development	3.580
		learning or memory	3.368
miR-3072-3p	2.01	synapse	11.804
		nervous system development	10.568
		synaptic signaling	10.263
		central nervous system development	6.600
		axon	6.092
		neuron spine	5.688
		learning or memory	4.474

**Table 2:** KEGG pathway analysis of genes regulated by miRNAs.

miRNA	KEGG Pathway	*p*-Value
miR-6240	Long-term potentiation	4.957 × 10^−4^
	Axon guidance	1.005 × 10^−3^
	cAMP signaling pathway	1.318 × 10^−3^
miR-1983	FoxO signaling pathway	6.262 × 10^−9^
	cAMP signaling pathway	1.010 × 10^−3^
	Calcium signaling pathway	1.287 × 10^−3^
	Long-term depression	1.767 × 10^−3^
	Axon guidance	2.549 × 10^−3^
	AMPK signaling pathway	9.073 × 10^−3^
	GABAergic synapse	2.809 × 10^−2^
miR-3963	Axon guidance	3.766 × 10^−3^
	cAMP signaling pathway	6.143 × 10^−3^
	MAPK signaling pathway	1.322 × 10^−2^
	Long-term potentiation	3.981 × 10^−2^
miR-3968	Axon guidance	1.285 × 10^−4^
	MAPK signaling pathway	5.188 × 10^−3^
	FoxO signaling pathway	1.429 × 10^−2^
	Circadian entrainment	3.075 × 10^−2^
miR-125a-3p	MAPK signaling pathway	1.032 × 10^−4^
	Dopaminergic synapse	1.213 × 10^−4^
	Axon guidance	2.967 × 10^−4^
	AMPK signaling pathway	1.233 × 10^−3^
	FoxO signaling pathway	2.631 × 10^−3^
	Long-term depression	2.756 × 10^−2^
	GABAergic synapse	3.667 × 10^−2^
miR-6366	FoxO signaling pathway	1.938 × 10^−7^
	Axon guidance	2.215 × 10^−6^
	MAPK signaling pathway	4.478 × 10^−6^
	cAMP signaling pathway	5.824 × 10^−5^
	Synaptic vesicle cycle	4.102 × 10^−3^
miR-3072-3p	Axon guidance	8.508 × 10^−5^
	cAMP signaling pathway	1.811 × 10^−2^
	FoxO signaling pathway	2.037 × 10^−2^
	MAPK signaling pathway	2.510 × 10^−2^
	AMPK signaling pathway	2.539 × 10^−2^

## Data Availability

The data that support the findings of this study are available from the corresponding author, Y.K., upon reasonable request.

## Supplementary Materials

The following supporting information can be downloaded at: https://www.mdpi.com/article/10.3390/nu15061479/s1, Table S1: Primers used for RT-qPCR; Figure S1: Specificity check of PCR reaction; Figure S2: C2C12 cells induced to differentiate in the presence of various concentrations of carnosine (0~50 mM).
