# Supplementary material for: Molecular Mechanisms for the Carnosine-Induced Activation of Muscle–Brain Interaction
Source: Nutrients. 2023 Mar 19;15(6):1479. doi: 10.3390/nu15061479 (PMC10057344; doi:10.3390/nu15061479)
Supplement: Supplementary file 1 [file nutrients-15-01479-s001.zip › nutrients-2297724-supplementary.pdf]

Table S1 Primers used for RT-qPCR

Primer	sequence
β-actin	GGCCAGGTCATCACTATTG
	GAGGTCTTTACGGATGTCAAC
MyoD	ATGAGGCCTTCGAGACGCTC
	CAGAGCCTGCAGACCTTCGA
Myogenin	CCTACAGGCCTTGCTCAGCT
	CGAACTCCAGTGCATTGCCC
MyHC1	AGCATTCTCCTGCTGTTTCCT
	GGCTGAGCCTTGGATTCTCA
MyHC2a	ATTCTCAGGCTTCAGGATTTGGTG
	CTTGCGGAACTTGGATAGATTTGTG
MyHC2x	CATCCCTAAGGCAGGCTCT
	AGCCTCGATTCGCTCCTTTT
MyHC2b	CTGCAGGACTTGGTGGACAAACTA
	TTGGCCAGGTTGACATTGGA
Sema3A	GGCCAGGTCATCACTATTG
	GAGGTCTTTACGGATGTCAAC
Netrin-1	GCCTTCCTCACCGACCTCAA
	TTCTTGCCGAGCGACAGAGT
Neuropilin 1	ACCCTGAGAGAGCCACACAC
	CCTGGTCGTCGTCACACTCA
Neuropilin 2	CTCTCGGGCCTCATTGCTGA
	GGCTTGAGGGTTCCGAGGAA
Neogenin	TCCCACTCCCATGATGCCAC
	TGGTGTTTGGGCAGGGAGTT
DCC	TTTGTCCGTTTGAGCTGGCG
	AAGCTCGCTCCCTGTTGTCA
Sirt1	GCAGACGTGGTAATGTCCAAACAG
	ACATCTTGGCAGTATTTGTGGTGAA
PGC-1α	CCGTAAATCTGCGGGATGATG
	CAGTTTCGTTCGACCTGCGTAA
TFAM	CATTTATCTATCTGAAAGCTTCC
	CTCTTCCCAAGACTTCATTTC
BDNF	AGTTGGAAGCCTGAATGAATGGA
	CTGATGCTCAGGAACCCAGGA
Irisin	TGCCCACATGAAGAGGACCA
	CCACGCGAGGCTGAAAAGAT
IL-15	CTCACCAGCAAGGACCATGAAG
	CCAGTTGCAGAGTTGGACGAAG

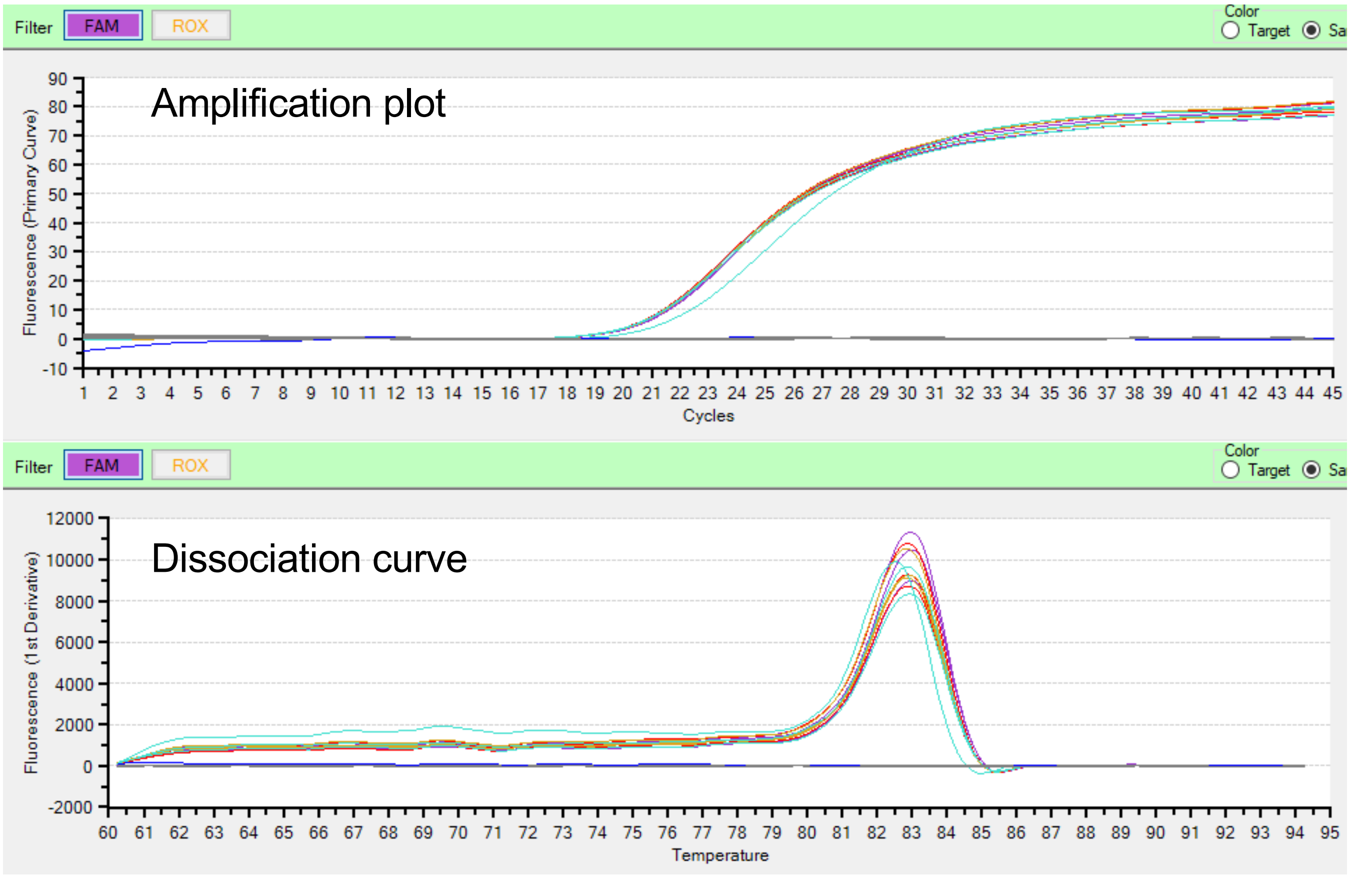

Figure. S1 Specificity check of PCR reaction. The specificity of the PCR reaction was confirmed by amplification plot and dissociation curve data for each gene.

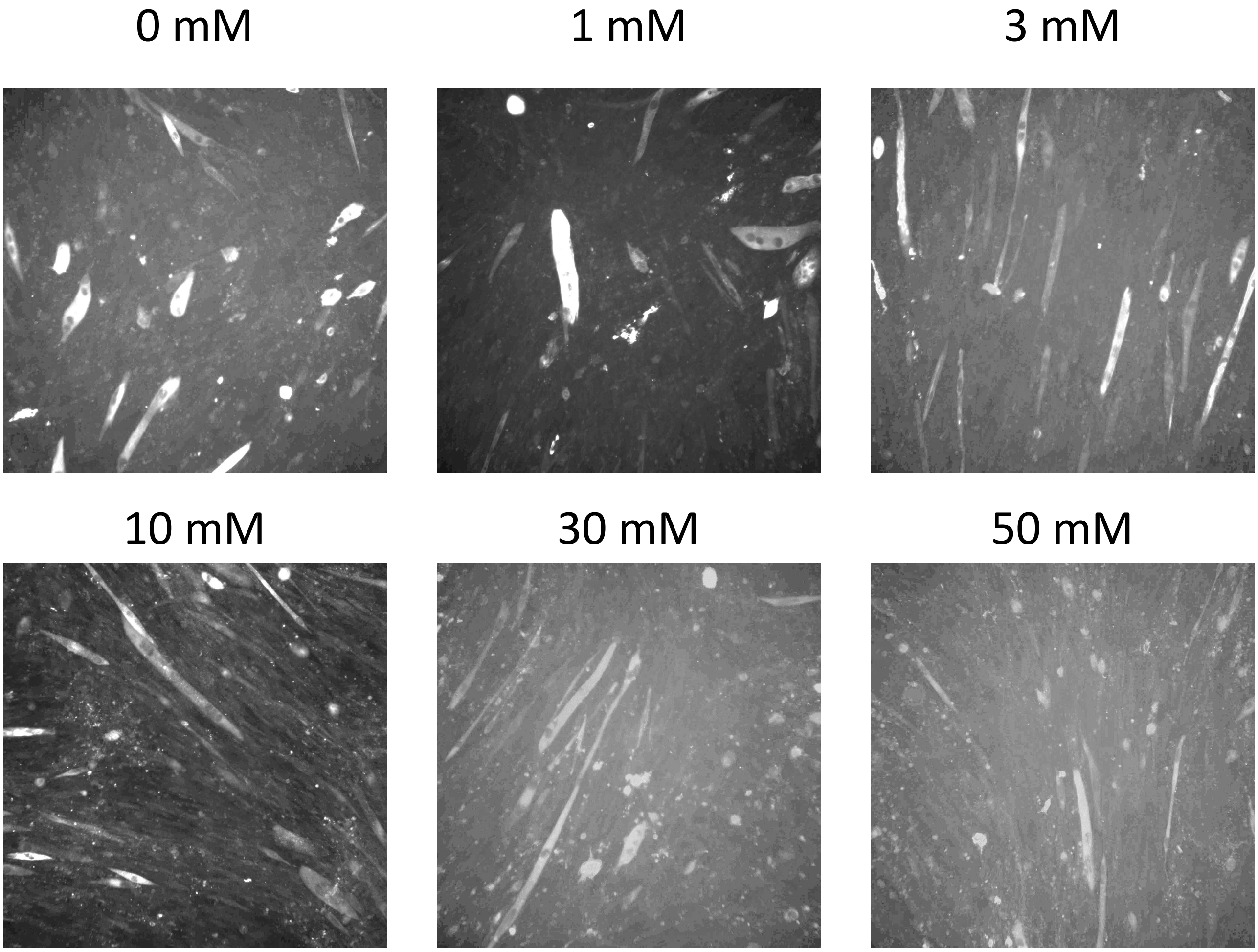

Figure. S2 C2C12 cells induced to differentiate in the presence of various concentrations of carnosine (0 ~ 50 mM). All stained cells are shown in grayscale, stained with slow and fast myosin skeletal heavy chain antibodies in C2C12 cells after differentiation. Same color photographs (0 mM and 50 mM) are also shown in Figure 2E and F.
